# Solitary giant molluscum contagiosum presenting as lid tumor in an immunocompetent child

**DOI:** 10.4103/0301-4738.62652

**Published:** 2010

**Authors:** Prem Vardhan, Shilpa Goel, Gaurav Goyal, Naresh Kumar

**Affiliations:** Guru Nanak Eye Centre, New Delhi, India; 1 Department of Medicine, Maulana Azad Medical College, New Delhi, India

**Keywords:** Giant molluscum contagiosum, lid tumor, solitary

## Abstract

We report the case of a three-year-old male child who presented with a single painless subcutaneously located upper lid mass of 1.2 × 1.5 cm with a central depression. The mass could be easily separated from overlying skin on complete excision biopsy and showed a never described before whitish brain like appearance consisting of multiple lobes and gyri, which histopathologically proved to be molluscum contagiosum (MC). Tests to investigate underlying immunodeficiency did not show any evidence of immunocompromised state.

Molluscum contagiosum (MC) is a skin infection caused by a member of the Poxvirus family, typically affecting young or immunocompromised persons.[1] It usually manifests as single or multiple umblicated papules or nodules on the skin and eyelid margin and less commonly on conjunctiva. We present a case of solitary giant MC unusually presenting as a large lid nodule of the upper eyelid in an immunocompetent three-year-old child.

## Case Report

A three-year-old child presented to our hospital with a single painless swelling over the left upper eyelid, which had been gradually increasing in size for the last five months. There was no history of trauma to the eye. He did not have any other swelling in his body. Fine needle aspiration cytology (FNAC) was done by a private practitioner two months before coming to this hospital and found to be inconclusive.

**Figure 1:**
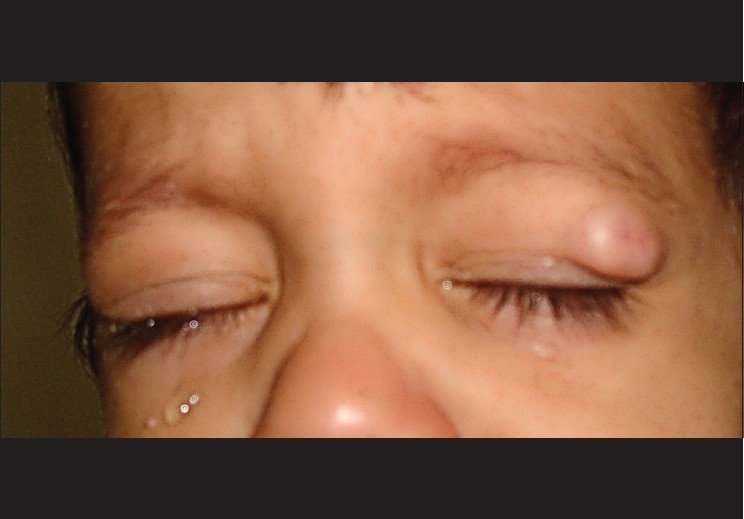
Solitary upper lid mass with central depression and scar

Examination revealed a single firm, nontender, and 1.2 × 1.5 cm mass over the outer upper half of the left upper eyelid [Fig. 1]. The overlying skin could not be pinched from the mass and there was an umblication in the center with a scar of needle prick and a central small opening, which produced nonpurulent whitish discharge on pressing the mass. The mass was freely mobile over the underlying lid. Differential diagnosis of sebaceous cyst, dermoid cyst, foreign body granuloma and cysticercosis was made and excision biopsy of mass was performed.

**Figure 2:**
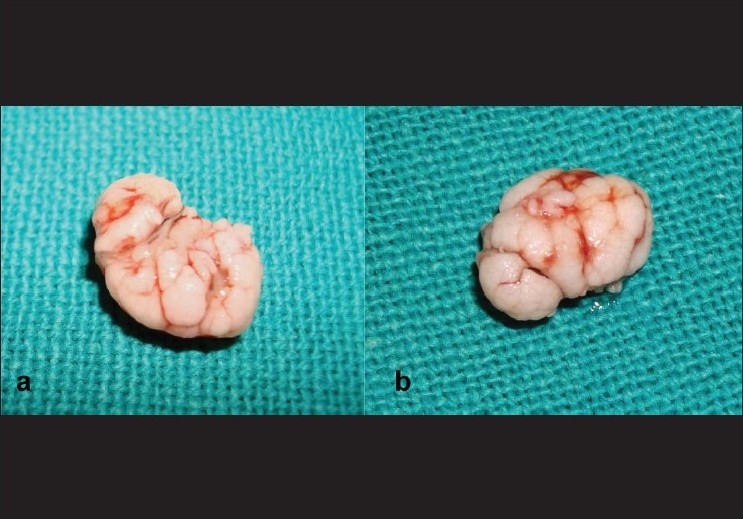
Peculiar whitish brain like appearance of excised mass showing surface corrugation (a) Anterior view (b) Posterior view

**Figure 3:**
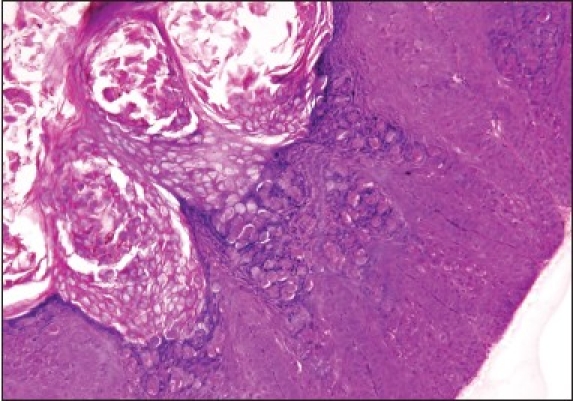
Epithelial hyperplasia with numerous intracytoplasmic inclusion bodies (H&E, ×100)

A skin incision was made along the lid crease, the skin was undermined over the mass which was unexpectedly easy in view of its adherence to mass on preoperative examination and the mass was easily separated from the underlying orbicularis. The mass was removed in toto and primary skin closure was done. The excised mass was white in color and, interestingly, had brain like appearance morphologically. It had multiple lobes and gyri like corrugations over its surface as shown in Fig. 2. Histopathology showed typical epithelial hyperplasia and intracytoplasmic inclusion bodies and confirmed the diagnosis of MC [Fig. 3]. No recurrence or evolution of new lesion was noted on one year follow-up.

Following the diagnosis of MC, patient was systematically reviewed and several investigations were done to look for immunosuppressive condition. He had hemoglobin of 15 gm%, total leukocyte count of 6700/mm^3^, platelet count of 3.5 lakhs/mm^3^ and the peripheral smear showed no atypical cell. He had blood urea of 26 mg/dl, serum creatinine of 0.6 mg/dl, serum bilirubin of 0.6mg/dL, SGOT and SGPT of 26 U/L and 32 U/L respectively, serum album of 5g/dl and serum globulin of 3 gm/dl. Serology for Human immunodeficiency Virus (HIV) and hepatitis B was negative in this patient, thus, excluding common causes of immunodeficiency.

## Discussion

In conditions with altered immunity such as atopic dermatitis,[2] corticosteroid and immunosuppressive therapy,[3] sarcoidosis, leukemias, Wiskott Aldrich syndrome and Acquired Immunodeficiency Syndrome (AIDS),[4,5] atypical lesions of MC may occur, often reaching a large size more than 1 cm^2^ and then it is called giant MC. Although there are many case reports of giant MC in immunocompetent individuals elsewhere on body, Medline search revealed the only one case report of giant MC involving the eyelid in an immunocompetent patient.[6]

A solitary giant molluscum contagiosum of eyelid can mimic commonly found lid tumors like sebaceous cyst, dermoid cyst and other skin tumors. The characteristic central umblication of MC persists even in its giant variant, which is an important clue to differentiate it from other skin tumors. The central scar with a small oozing opening present in this case was probably due to a previously done fine needle aspiration cytology.

Complete excision of the mass provided us with a peculiar white brain like appearance of the mass having multiple lobes and surface corrugations [Fig. 2], which to the best of our knowledge has not been described in literature so far. Authors feel that this gross pathological appearance of mass is quite distinct and if found in any case could point towards the diagnosis of MC. No recurrence has been observed over one year period of follow-up.

Various other described treatment modalities for MC include cryotherapy with liquid nitrogen, extirpation followed by cauterization of the base with electro-dessication, or chemical agents such as silver nitrate, phenol and trichloroacetic acid. Resistant cases in immunocompromised patients have been treated with topical antiviral agent cidofovir (5%) or intralesional and systemic interferon alpha, imiquimod 5% cream.

## Conclusion

Solitary giant MC, although a rare condition in immunocompetent patients, should be suspected if central umbilication is found in the lid tumor. Complete excision of the mass is an easy and effective mode of treatment and white brain like appearance of the removed tissue may further point towards the diagnosis of MC.
